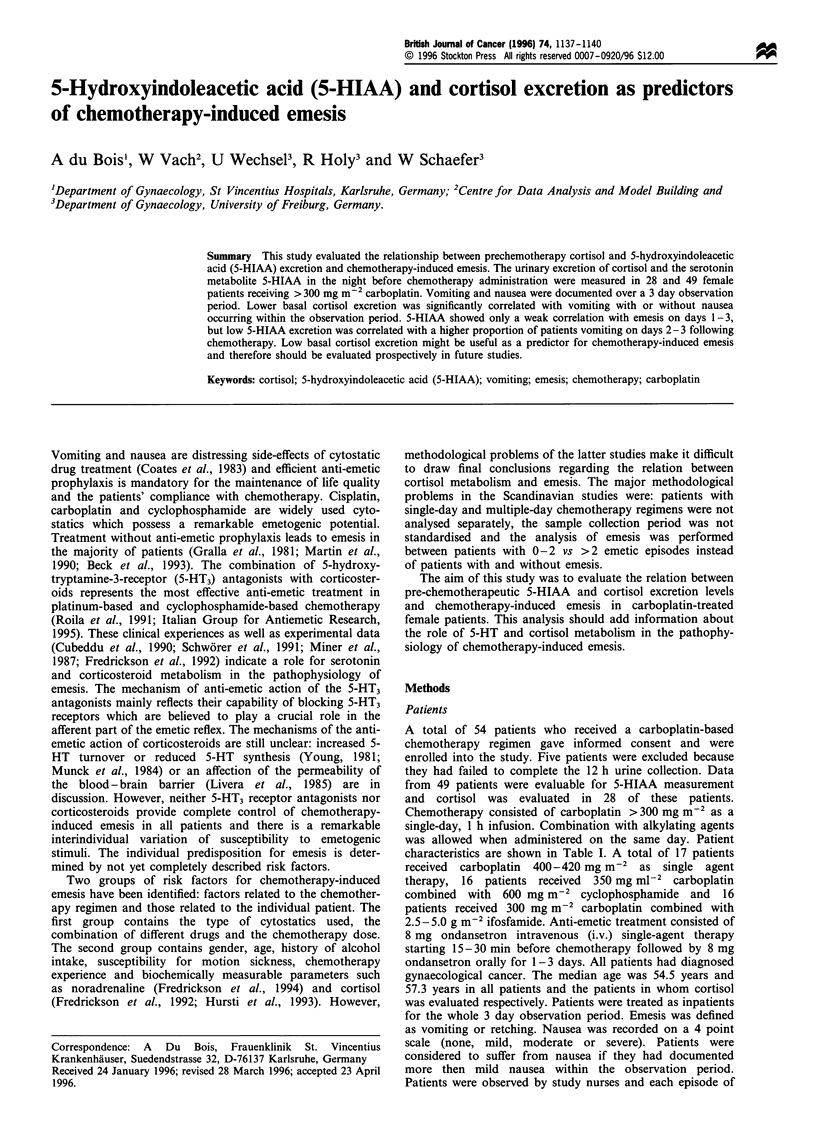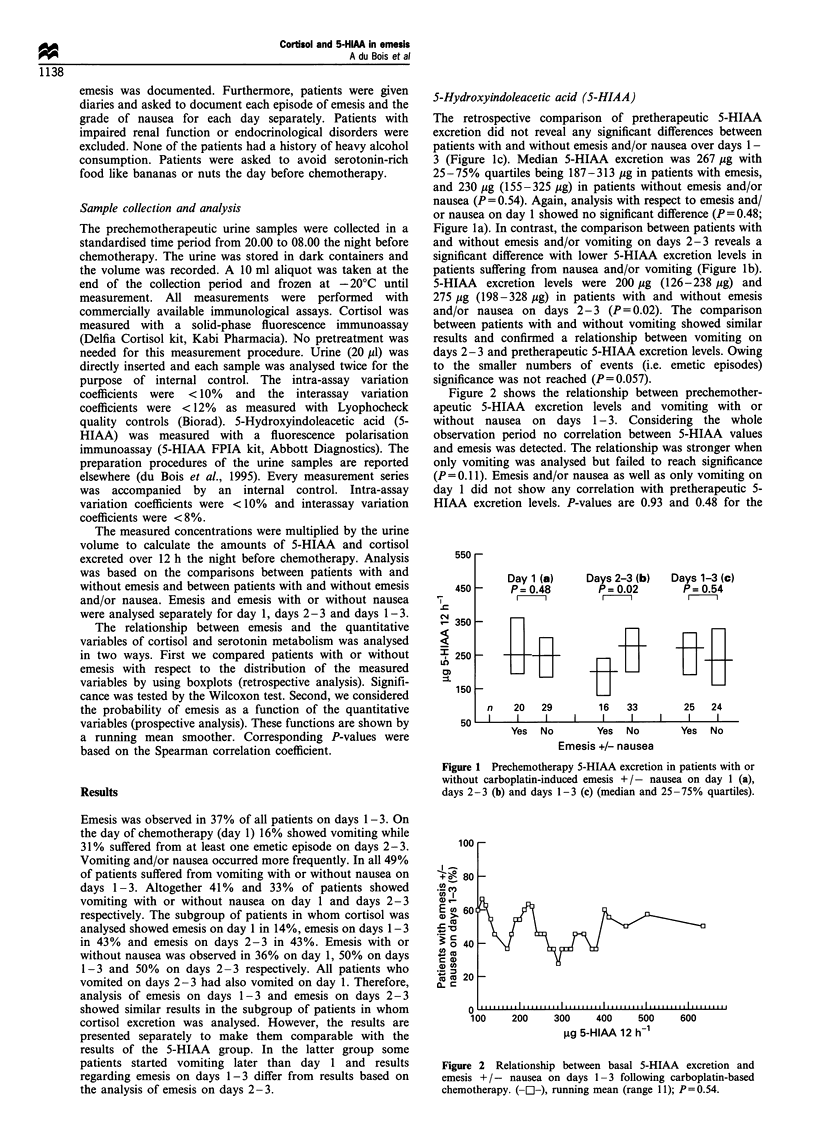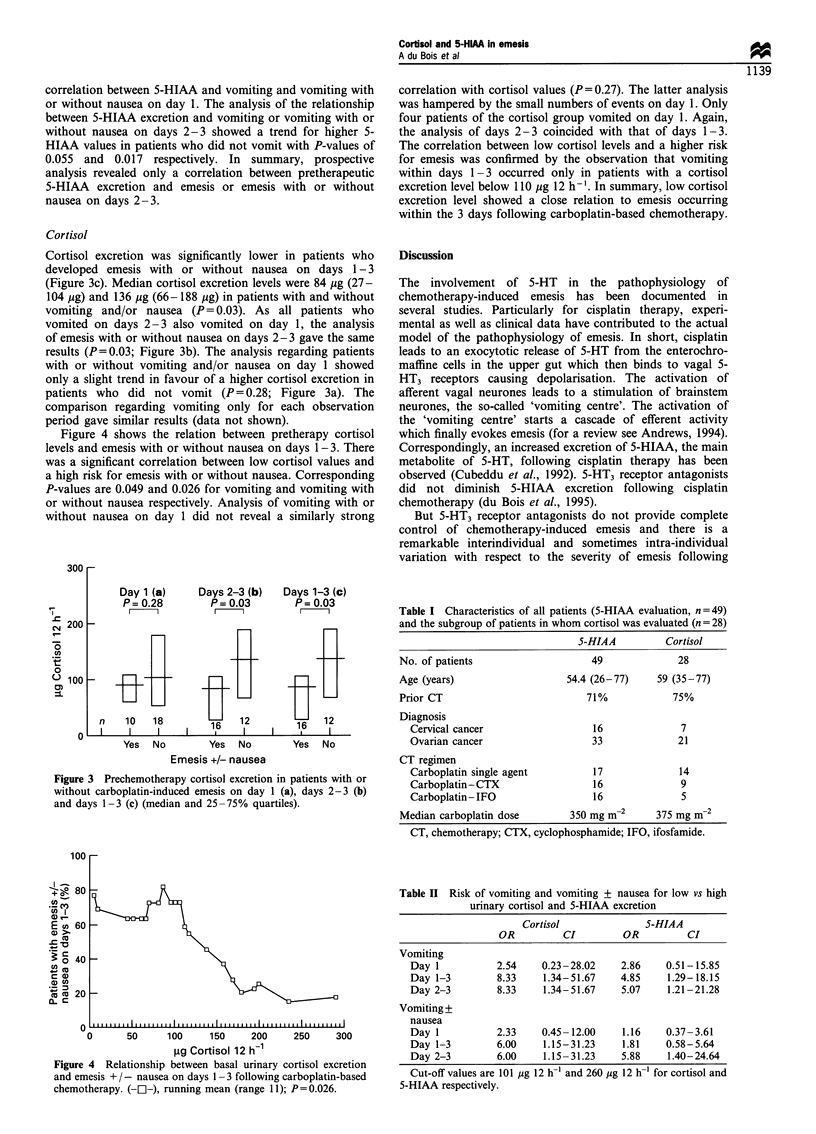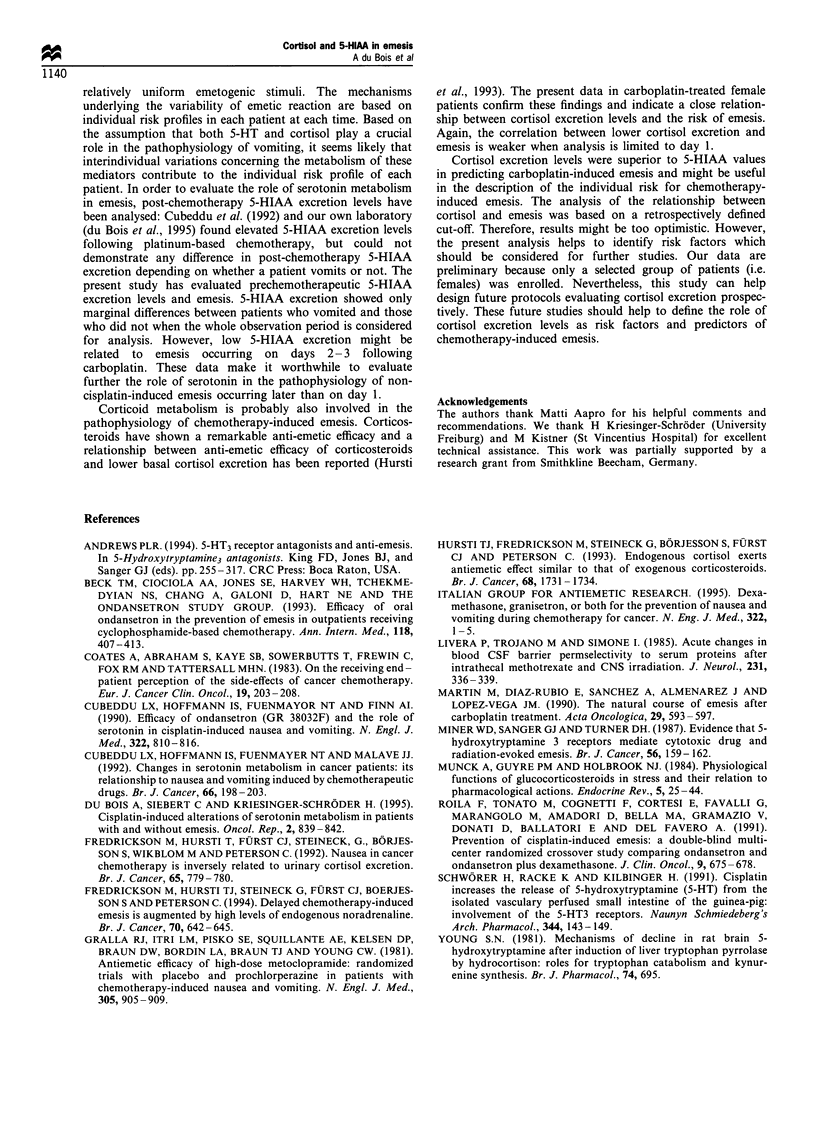# 5-Hydroxyindoleacetic acid (5-HIAA) and cortisol excretion as predictors of chemotherapy-induced emesis.

**DOI:** 10.1038/bjc.1996.503

**Published:** 1996-10

**Authors:** A. du Bois, W. Vach, U. Wechsel, R. Holy, W. Schaefer

**Affiliations:** Department of Gynaecology, St Vincentius Hospitals, Karlsruhe, Germany.

## Abstract

This study evaluated the relationship between prechemotherapy cortisol and 5-hydroxyindoleacetic acid (5-HIAA) excretion and chemotherapy-induced emesis. The urinary excretion of cortisol and the serotonin metabolite 5-HIAA in the night before chemotherapy administration were measured in 28 and 49 female patients receiving > 300 mg m-2 carboplatin. Vomiting and nausea were documented over a 3 day observation period. Lower basal cortisol excretion was significantly correlated with vomiting with or without nausea occurring within the observation period. 5-HIAA showed only a weak correlation with emesis on days 1-3, but low 5-HIAA excretion was correlated with a higher proportion of patients vomiting on days 2-3 following chemotherapy. Low basal cortisol excretion might be useful as a predictor for chemotherapy-induced emesis and therefore should be evaluated prospectively in future studies.


British Journal of Cancer (1996) 74, 1137-1140
© 1996 Stockton Press All rights reserved 0007-0920/96 $12.00

### 5-Hydroxyindoleacetic acid (5-HIAA) and cortisol excretion as predictors of chemotherapy-induced emesis

A du Bois[1], W Vach[2], U Wechsel[3], R Holy[3] and W Schaefer[3]

[1]*Department of Gynaecology, St Vincentius Hospitals, Karlsruhe, Germany;* [2]*Centre for Data Analysis and Model Building and* [3]*Department of Gynaecology, University of Freiburg, Germany.*

**Summary** This study evaluated the relationship between prechemotherapy cortisol and 5-hydroxyindoleacetic acid (5-HIAA) excretion and chemotherapy-induced emesis. The urinary excretion of cortisol and the serotonin metabolite 5-HIAA in the night before chemotherapy administration were measured in 28 and 49 female patients receiving $>300$ mg m$^{-2}$ carboplatin. Vomiting and nausea were documented over a 3 day observation period. Lower basal cortisol excretion was significantly correlated with vomiting with or without nausea occurring within the observation period. 5-HIAA showed only a weak correlation with emesis on days 1-3, but low 5-HIAA excretion was correlated with a higher proportion of patients vomiting on days 2-3 following chemotherapy. Low basal cortisol excretion might be useful as a predictor for chemotherapy-induced emesis and therefore should be evaluated prospectively in future studies.

**Keywords:** cortisol; 5-hydroxyindoleacetic acid (5-HIAA); vomiting; emesis; chemotherapy; carboplatin

Vomiting and nausea are distressing side-effects of cytostatic drug treatment (Coates *et al.*, 1983) and efficient anti-emetic prophylaxis is mandatory for the maintenance of life quality and the patients' compliance with chemotherapy. Cisplatin, carboplatin and cyclophosphamide are widely used cytostatics which possess a remarkable emetogenic potential. Treatment without anti-emetic prophylaxis leads to emesis in the majority of patients (Gralla *et al.*, 1981; Martin *et al.*, 1990; Beck *et al.*, 1993). The combination of 5-hydroxytryptamine-3-receptor ($5\text{-HT}_3$) antagonists with corticosteroids represents the most effective anti-emetic treatment in platinum-based and cyclophosphamide-based chemotherapy (Roila *et al.*, 1991; Italian Group for Antiemetic Research, 1995). These clinical experiences as well as experimental data (Cubeddu *et al.*, 1990; Schwörer *et al.*, 1991; Miner *et al.*, 1987; Fredrickson *et al.*, 1992) indicate a role for serotonin and corticosteroid metabolism in the pathophysiology of emesis. The mechanism of anti-emetic action of the $5\text{-HT}_3$ antagonists mainly reflects their capability of blocking $5\text{-HT}_3$ receptors which are believed to play a crucial role in the afferent part of the emetic reflex. The mechanisms of the anti-emetic action of corticosteroids are still unclear: increased 5-HT turnover or reduced 5-HT synthesis (Young, 1981; Munck *et al.*, 1984) or an affection of the permeability of the blood-brain barrier (Livera *et al.*, 1985) are in discussion. However, neither $5\text{-HT}_3$ receptor antagonists nor corticosteroids provide complete control of chemotherapy-induced emesis in all patients and there is a remarkable interindividual variation of susceptibility to emetogenic stimuli. The individual predisposition for emesis is determined by not yet completely described risk factors.

Two groups of risk factors for chemotherapy-induced emesis have been identified: factors related to the chemotherapy regimen and those related to the individual patient. The first group contains the type of cytostatics used, the combination of different drugs and the chemotherapy dose. The second group contains gender, age, history of alcohol intake, susceptibility for motion sickness, chemotherapy experience and biochemically measurable parameters such as noradrenaline (Fredrickson *et al.*, 1994) and cortisol (Fredrickson *et al.*, 1992; Hursti *et al.*, 1993). However, methodological problems of the latter studies make it difficult to draw final conclusions regarding the relation between cortisol metabolism and emesis. The major methodological problems in the Scandinavian studies were: patients with single-day and multiple-day chemotherapy regimens were not analysed separately, the sample collection period was not standardised and the analysis of emesis was performed between patients with 0-2 *vs* >2 emetic episodes instead of patients with and without emesis.

The aim of this study was to evaluate the relation between pre-chemotherapeutic 5-HIAA and cortisol excretion levels and chemotherapy-induced emesis in carboplatin-treated female patients. This analysis should add information about the role of 5-HT and cortisol metabolism in the pathophysiology of chemotherapy-induced emesis.

### Methods

### *Patients*

A total of 54 patients who received a carboplatin-based chemotherapy regimen gave informed consent and were enrolled into the study. Five patients were excluded because they had failed to complete the 12 h urine collection. Data from 49 patients were evaluable for 5-HIAA measurement and cortisol was evaluated in 28 of these patients. Chemotherapy consisted of carboplatin $>300$ mg m$^{-2}$ as a single-day, 1 h infusion. Combination with alkylating agents was allowed when administered on the same day. Patient characteristics are shown in Table I. A total of 17 patients received carboplatin 400-420 mg m$^{-2}$ as single agent therapy, 16 patients received 350 mg ml$^{-2}$ carboplatin combined with 600 mg m$^{-2}$ cyclophosphamide and 16 patients received 300 mg m$^{-2}$ carboplatin combined with 2.5-5.0 g m$^{-2}$ ifosfamide. Anti-emetic treatment consisted of 8 mg ondansetron intravenous (i.v.) single-agent therapy starting 15-30 min before chemotherapy followed by 8 mg ondansetron orally for 1-3 days. All patients had diagnosed gynaecological cancer. The median age was 54.5 years and 57.3 years in all patients and the patients in whom cortisol was evaluated respectively. Patients were treated as inpatients for the whole 3 day observation period. Emesis was defined as vomiting or retching. Nausea was recorded on a 4 point scale (none, mild, moderate or severe). Patients were considered to suffer from nausea if they had documented more then mild nausea within the observation period. Patients were observed by study nurses and each episode of

Correspondence: A Du Bois, Frauenklinik St. Vincentius Krankenhäuser, Suedendstrasse 32, D-76137 Karlsruhe, Germany
Received 24 January 1996; revised 28 March 1996; accepted 23 April 1996.

emesis was documented. Furthermore, patients were given diaries and asked to document each episode of emesis and the grade of nausea for each day separately. Patients with impaired renal function or endocrinological disorders were excluded. None of the patients had a history of heavy alcohol consumption. Patients were asked to avoid serotonin-rich food like bananas or nuts the day before chemotherapy.

*Sample collection and analysis*

The prechemotherapeutic urine samples were collected in a standardised time period from 20.00 to 08.00 the night before chemotherapy. The urine was stored in dark containers and the volume was recorded. A 10 ml aliquot was taken at the end of the collection period and frozen at −20°C until measurement. All measurements were performed with commercially available immunological assays. Cortisol was measured with a solid-phase fluorescence immunoassay (Delfia Cortisol kit, Kabi Pharmacia). No pretreatment was needed for this measurement procedure. Urine (20 μl) was directly inserted and each sample was analysed twice for the purpose of internal control. The intra-assay variation coefficients were <10% and the interassay variation coefficients were <12% as measured with Lyophocheck quality controls (Biorad). 5-Hydroxyindoleacetic acid (5-HIAA) was measured with a fluorescence polarisation immunoassay (5-HIAA FPIA kit, Abbott Diagnostics). The preparation procedures of the urine samples are reported elsewhere (du Bois *et al.*, 1995). Every measurement series was accompanied by an internal control. Intra-assay variation coefficients were <10% and interassay variation coefficients were <8%.

The measured concentrations were multiplied by the urine volume to calculate the amounts of 5-HIAA and cortisol excreted over 12 h the night before chemotherapy. Analysis was based on the comparisons between patients with and without emesis and between patients with and without emesis and/or nausea. Emesis and emesis with or without nausea were analysed separately for day 1, days 2–3 and days 1–3.

The relationship between emesis and the quantitative variables of cortisol and serotonin metabolism was analysed in two ways. First we compared patients with or without emesis with respect to the distribution of the measured variables by using boxplots (retrospective analysis). Significance was tested by the Wilcoxon test. Second, we considered the probability of emesis as a function of the quantitative variables (prospective analysis). These functions are shown by a running mean smoother. Corresponding *P*-values were based on the Spearman correlation coefficient.

### Results

Emesis was observed in 37% of all patients on days 1–3. On the day of chemotherapy (day 1) 16% showed vomiting while 31% suffered from at least one emetic episode on days 2–3. Vomiting and/or nausea occurred more frequently. In all 49% of patients suffered from vomiting with or without nausea on days 1–3. Altogether 41% and 33% of patients showed vomiting with or without nausea on day 1 and days 2–3 respectively. The subgroup of patients in whom cortisol was analysed showed emesis on day 1 in 14%, emesis on days 1–3 in 43% and emesis on days 2–3 in 43%. Emesis with or without nausea was observed in 36% on day 1, 50% on days 1–3 and 50% on days 2–3 respectively. All patients who vomited on days 2–3 had also vomited on day 1. Therefore, analysis of emesis on days 1–3 and emesis on days 2–3 showed similar results in the subgroup of patients in whom cortisol excretion was analysed. However, the results are presented separately to make them comparable with the results of the 5-HIAA group. In the latter group some patients started vomiting later than day 1 and results regarding emesis on days 1–3 differ from results based on the analysis of emesis on days 2–3.

*5-Hydroxyindoleacetic acid (5-HIAA)*

The retrospective comparison of pretherapeutic 5-HIAA excretion did not reveal any significant differences between patients with and without emesis and/or nausea over days 1–3 (Figure 1c). Median 5-HIAA excretion was 267 μg with 25–75% quartiles being 187–313 μg in patients with emesis, and 230 μg (155–325 μg) in patients without emesis and/or nausea ($P=0.54$). Again, analysis with respect to emesis and/or nausea on day 1 showed no significant difference ($P=0.48$; Figure 1a). In contrast, the comparison between patients with and without emesis and/or vomiting on days 2–3 reveals a significant difference with lower 5-HIAA excretion levels in patients suffering from nausea and/or vomiting (Figure 1b). 5-HIAA excretion levels were 200 μg (126–238 μg) and 275 μg (198–328 μg) in patients with and without emesis and/or nausea on days 2–3 ($P=0.02$). The comparison between patients with and without vomiting showed similar results and confirmed a relationship between vomiting on days 2–3 and pretherapeutic 5-HIAA excretion levels. Owing to the smaller numbers of events (i.e. emetic episodes) significance was not reached ($P=0.057$).

Figure 2 shows the relationship between prechemotherapeutic 5-HIAA excretion levels and vomiting with or without nausea on days 1–3. Considering the whole observation period no correlation between 5-HIAA values and emesis was detected. The relationship was stronger when only vomiting was analysed but failed to reach significance ($P=0.11$). Emesis and/or nausea as well as only vomiting on day 1 did not show any correlation with pretherapeutic 5-HIAA excretion levels. *P*-values are 0.93 and 0.48 for the

**Figure 1** Prechemotherapy 5-HIAA excretion in patients with or without carboplatin-induced emesis +/− nausea on day 1 (**a**), days 2–3 (**b**) and days 1–3 (**c**) (median and 25–75% quartiles).

**Figure 2** Relationship between basal 5-HIAA excretion and emesis +/− nausea on days 1–3 following carboplatin-based chemotherapy. (–□–), running mean (range 11); $P=0.54$.

correlation between 5-HIAA and vomiting and vomiting with or without nausea on day 1. The analysis of the relationship between 5-HIAA excretion and vomiting or vomiting with or without nausea on days 2–3 showed a trend for higher 5-HIAA values in patients who did not vomit with *P*-values of 0.055 and 0.017 respectively. In summary, prospective analysis revealed only a correlation between pretherapeutic 5-HIAA excretion and emesis or emesis with or without nausea on days 2–3.

### *Cortisol*

Cortisol excretion was significantly lower in patients who developed emesis with or without nausea on days 1–3 (Figure 3c). Median cortisol excretion levels were 84 $\mu g$ (27–104 $\mu g$) and 136 $\mu g$ (66–188 $\mu g$) in patients with and without vomiting and/or nausea ($P = 0.03$). As all patients who vomited on days 2–3 also vomited on day 1, the analysis of emesis with or without nausea on days 2–3 gave the same results ($P = 0.03$; Figure 3b). The analysis regarding patients with or without vomiting and/or nausea on day 1 showed only a slight trend in favour of a higher cortisol excretion in patients who did not vomit ($P = 0.28$; Figure 3a). The comparison regarding vomiting only for each observation period gave similar results (data not shown).

Figure 4 shows the relation between pretherapy cortisol levels and emesis with or without nausea on days 1–3. There was a significant correlation between low cortisol values and a high risk for emesis with or without nausea. Corresponding *P*-values are 0.049 and 0.026 for vomiting and vomiting with or without nausea respectively. Analysis of vomiting with or without nausea on day 1 did not reveal a similarly strong correlation with cortisol values ($P = 0.27$). The latter analysis was hampered by the small numbers of events on day 1. Only four patients of the cortisol group vomited on day 1. Again, the analysis of days 2–3 coincided with that of days 1–3. The correlation between low cortisol levels and a higher risk for emesis was confirmed by the observation that vomiting within days 1–3 occurred only in patients with a cortisol excretion level below 110 $\mu g$ 12 $h^{-1}$. In summary, low cortisol excretion level showed a close relation to emesis occurring within the 3 days following carboplatin-based chemotherapy.

### Discussion

The involvement of 5-HT in the pathophysiology of chemotherapy-induced emesis has been documented in several studies. Particularly for cisplatin therapy, experimental as well as clinical data have contributed to the actual model of the pathophysiology of emesis. In short, cisplatin leads to an exocytotic release of 5-HT from the enterochromaffine cells in the upper gut which then binds to vagal 5-$HT_3$ receptors causing depolarisation. The activation of afferent vagal neurones leads to a stimulation of brainstem neurones, the so-called 'vomiting centre'. The activation of the 'vomiting centre' starts a cascade of efferent activity which finally evokes emesis (for a review see Andrews, 1994). Correspondingly, an increased excretion of 5-HIAA, the main metabolite of 5-HT, following cisplatin therapy has been observed (Cubeddu *et al.*, 1992). 5-$HT_3$ receptor antagonists did not diminish 5-HIAA excretion following cisplatin chemotherapy (du Bois *et al.*, 1995).

But 5-$HT_3$ receptor antagonists do not provide complete control of chemotherapy-induced emesis and there is a remarkable interindividual and sometimes intra-individual variation with respect to the severity of emesis following

**Figure 3** Prechemotherapy cortisol excretion in patients with or without carboplatin-induced emesis on day 1 (**a**), days 2–3 (**b**) and days 1–3 (**c**) (median and 25–75% quartiles).

**Figure 4** Relationship between basal urinary cortisol excretion and emesis +/– nausea on days 1–3 following carboplatin-based chemotherapy. (-□-), running mean (range 11); $P = 0.026$.

**Table I** Characteristics of all patients (5-HIAA evaluation, $n = 49$) and the subgroup of patients in whom cortisol was evaluated ($n = 28$)

| | *5-HIAA* | *Cortisol* |
|---|---|---|
| No. of patients | 49 | 28 |
| Age (years) | 54.4 (26–77) | 59 (35–77) |
| Prior CT | 71% | 75% |
| Diagnosis | | |
| Cervical cancer | 16 | 7 |
| Ovarian cancer | 33 | 21 |
| CT regimen | | |
| Carboplatin single agent | 17 | 14 |
| Carboplatin–CTX | 16 | 9 |
| Carboplatin–IFO | 16 | 5 |
| Median carboplatin dose | 350 mg $m^{-2}$ | 375 mg $m^{-2}$ |

CT, chemotherapy; CTX, cyclophosphamide; IFO, ifosfamide.

**Table II** Risk of vomiting and vomiting ± nausea for low *vs* high urinary cortisol and 5-HIAA excretion

| | *Cortisol* | | *5-HIAA* | |
|---|---|---|---|---|
| | *OR* | *CI* | *OR* | *CI* |
| Vomiting | | | | |
| Day 1 | 2.54 | 0.23–28.02 | 2.86 | 0.51–15.85 |
| Day 1-3 | 8.33 | 1.34–51.67 | 4.85 | 1.29–18.15 |
| Day 2-3 | 8.33 | 1.34–51.67 | 5.07 | 1.21–21.28 |
| Vomiting ± nausea | | | | |
| Day 1 | 2.33 | 0.45–12.00 | 1.16 | 0.37–3.61 |
| Day 1-3 | 6.00 | 1.15–31.23 | 1.81 | 0.58–5.64 |
| Day 2-3 | 6.00 | 1.15–31.23 | 5.88 | 1.40–24.64 |

Cut-off values are 101 $\mu g$ 12 $h^{-1}$ and 260 $\mu g$ 12 $h^{-1}$ for cortisol and 5-HIAA respectively.

relatively uniform emetogenic stimuli. The mechanisms underlying the variability of emetic reaction are based on individual risk profiles in each patient at each time. Based on the assumption that both 5-HT and cortisol play a crucial role in the pathophysiology of vomiting, it seems likely that interindividual variations concerning the metabolism of these mediators contribute to the individual risk profile of each patient. In order to evaluate the role of serotonin metabolism in emesis, post-chemotherapy 5-HIAA excretion levels have been analysed: Cubeddu *et al.* (1992) and our own laboratory (du Bois *et al.*, 1995) found elevated 5-HIAA excretion levels following platinum-based chemotherapy, but could not demonstrate any difference in post-chemotherapy 5-HIAA excretion depending on whether a patient vomits or not. The present study has evaluated prechemotherapeutic 5-HIAA excretion levels and emesis. 5-HIAA excretion showed only marginal differences between patients who vomited and those who did not when the whole observation period is considered for analysis. However, low 5-HIAA excretion might be related to emesis occurring on days 2–3 following carboplatin. These data make it worthwhile to evaluate further the role of serotonin in the pathophysiology of non-cisplatin-induced emesis occurring later than on day 1.

Corticoid metabolism is probably also involved in the pathophysiology of chemotherapy-induced emesis. Corticosteroids have shown a remarkable anti-emetic efficacy and a relationship between anti-emetic efficacy of corticosteroids and lower basal cortisol excretion has been reported (Hursti *et al.*, 1993). The present data in carboplatin-treated female patients confirm these findings and indicate a close relationship between cortisol excretion levels and the risk of emesis. Again, the correlation between lower cortisol excretion and emesis is weaker when analysis is limited to day 1.

Cortisol excretion levels were superior to 5-HIAA values in predicting carboplatin-induced emesis and might be useful in the description of the individual risk for chemotherapy-induced emesis. The analysis of the relationship between cortisol and emesis was based on a retrospectively defined cut-off. Therefore, results might be too optimistic. However, the present analysis helps to identify risk factors which should be considered for further studies. Our data are preliminary because only a selected group of patients (i.e. females) was enrolled. Nevertheless, this study can help design future protocols evaluating cortisol excretion prospectively. These future studies should help to define the role of cortisol excretion levels as risk factors and predictors of chemotherapy-induced emesis.

**Acknowledgements**
The authors thank Matti Aapro for his helpful comments and recommendations. We thank H Kriesinger-Schröder (University Freiburg) and M Kistner (St Vincentius Hospital) for excellent technical assistance. This work was partially supported by a research grant from Smithkline Beecham, Germany.

### References

ANDREWS PLR. (1994). 5-$HT_3$ receptor antagonists and anti-emesis. In *5-Hydroxytryptamine₃ antagonists*. King FD, Jones BJ, and Sanger GJ (eds). pp. 255–317. CRC Press: Boca Raton, USA.

BECK TM, CIOCIOLA AA, JONES SE, HARVEY WH, TCHEKMEDYIAN NS, CHANG A, GALONI D, HART NE AND THE ONDANSETRON STUDY GROUP. (1993). Efficacy of oral ondansetron in the prevention of emesis in outpatients receiving cyclophosphamide-based chemotherapy. *Ann. Intern. Med.*, **118**, 407–413.

COATES A, ABRAHAM S, KAYE SB, SOWERBUTTS T, FREWIN C, FOX RM AND TATTERSALL MHN. (1983). On the receiving end – patient perception of the side-effects of cancer chemotherapy. *Eur. J. Cancer Clin. Oncol.*, **19**, 203–208.

CUBEDDU LX, HOFFMANN IS, FUENMAYOR NT AND FINN AI. (1990). Efficacy of ondansetron (GR 38032F) and the role of serotonin in cisplatin-induced nausea and vomiting. *N. Engl. J. Med.*, **322**, 810–816.

CUBEDDU LX, HOFFMANN IS, FUENMAYOR NT AND MALAVE JJ. (1992). Changes in serotonin metabolism in cancer patients: its relationship to nausea and vomiting induced by chemotherapeutic drugs. *Br. J. Cancer*, **66**, 198–203.

DU BOIS A, SIEBERT C AND KRIESINGER-SCHRÖDER H. (1995). Cisplatin-induced alterations of serotonin metabolism in patients with and without emesis. *Oncol. Rep.*, **2**, 839–842.

FREDRICKSON M, HURSTI T, FÜRST CJ, STEINECK, G., BÖRJESSON S, WIKBLOM M AND PETERSON C. (1992). Nausea in cancer chemotherapy is inversely related to urinary cortisol excretion. *Br. J. Cancer*, **65**, 779–780.

FREDRICKSON M, HURSTI TJ, STEINECK G, FÜRST CJ, BOERJESSON S AND PETERSON C. (1994). Delayed chemotherapy-induced emesis is augmented by high levels of endogenous noradrenaline. *Br. J. Cancer*, **70**, 642–645.

GRALLA RJ, ITRI LM, PISKO SE, SQUILLANTE AE, KELSEN DP, BRAUN DW, BORDIN LA, BRAUN TJ AND YOUNG CW. (1981). Antiemetic efficacy of high-dose metoclopramide: randomized trials with placebo and prochlorperazine in patients with chemotherapy-induced nausea and vomiting. *N. Engl. J. Med.*, **305**, 905–909.

HURSTI TJ, FREDRICKSON M, STEINECK G, BÖRJESSON S, FÜRST CJ AND PETERSON C. (1993). Endogenous cortisol exerts antiemetic effect similar to that of exogenous corticosteroids. *Br. J. Cancer*, **68**, 1731–1734.

ITALIAN GROUP FOR ANTIEMETIC RESEARCH. (1995). Dexamethasone, granisetron, or both for the prevention of nausea and vomiting during chemotherapy for cancer. *N. Eng. J. Med.*, **322**, 1–5.

LIVERA P, TROJANO M AND SIMONE I. (1985). Acute changes in blood CSF barrier permselectivity to serum proteins after intrathecal methotrexate and CNS irradiation. *J. Neurol.*, **231**, 336–339.

MARTIN M, DIAZ-RUBIO E, SANCHEZ A, ALMENAREZ J AND LOPEZ-VEGA JM. (1990). The natural course of emesis after carboplatin treatment. *Acta Oncologica*, **29**, 593–597.

MINER WD, SANGER GJ AND TURNER DH. (1987). Evidence that 5-hydroxytryptamine 3 receptors mediate cytotoxic drug and radiation-evoked emesis. *Br. J. Cancer*, **56**, 159–162.

MUNCK A, GUYRE PM AND HOLBROOK NJ. (1984). Physiological functions of glucocorticosteroids in stress and their relation to pharmacological actions. *Endocrine Rev.*, **5**, 25–44.

ROILA F, TONATO M, COGNETTI F, CORTESI E, FAVALLI G, MARANGOLO M, AMADORI D, BELLA MA, GRAMAZIO V, DONATI D, BALLATORI E AND DEL FAVERO A. (1991). Prevention of cisplatin-induced emesis: a double-blind multicenter randomized crossover study comparing ondansetron and ondansetron plus dexamethasone. *J. Clin. Oncol.*, **9**, 675–678.

SCHWÖRER H, RACKE K AND KILBINGER H. (1991). Cisplatin increases the release of 5-hydroxytryptamine (5-HT) from the isolated vasculary perfused small intestine of the guinea-pig: involvement of the 5-HT3 receptors. *Naunyn Schmiedeberg's Arch. Pharmacol.*, **344**, 143–149.

YOUNG S.N. (1981). Mechanisms of decline in rat brain 5-hydroxytryptamine after induction of liver tryptophan pyrrolase by hydrocortison: roles for tryptophan catabolism and kynurenine synthesis. *Br. J. Pharmacol.*, **74**, 695.